# Preparation and Properties of Fluorosilicone Fouling-Release Coatings

**DOI:** 10.3390/polym14183804

**Published:** 2022-09-11

**Authors:** Tong Wu, Yuhong Qi, Qi’an Chen, Chuanjun Gu, Zhanping Zhang

**Affiliations:** Department of Materials Science and Engineering, Dalian Maritime University, Dalian 116026, China

**Keywords:** fluorosilicone, hydroxyl terminated fluoropolysiloxane, antifouling, silicone oil, coatings

## Abstract

To improve the antifouling performance of silicone fouling-release coatings, some fluorosilicone and silicone fouling-release coatings were prepared and cured at room temperature with hydroxyl-terminated fluoropolysiloxane (FPS) or hydroxy-terminated polydimethylsiloxane (PDMS) as a film-forming resin, tetraethyl orthosilicate (TEOS) as a crosslinking agent, and dibutyltin dilaurate (DBTDL) as a catalyst. The chemical structure, surface morphology and roughness, tensile properties, and antifouling properties of the coating were studied by infrared spectroscopy, a laser confocal scanning microscope, contact angle measurement, tensile tests, and marine bacteria and benthic diatom attachment tests. The results showed that the FPS coatings were not only hydrophobic but also oleophobic, and the contact angles of the FPS coatings were larger than those of the PDMS coatings. The surface free energies of the FPS coatings were much lower than those of the PDMS coatings. Generally, the fluorine groups can improve the antifouling performance of the coating. Introducing nonreactive silicone oil into PDMS or FPS coatings can improve the antifouling performance of the coating to a certain extent. The prepared fluorosilicone fouling-release coatings showed good application prospects.

## 1. Introduction

Marine organisms such as microorganisms, barnacles, and algae adhere to the surface of ships and accumulate, resulting in an irregular distribution [1] and marine biological pollution. If the marine biological pollution is not controlled, the ships’ surface roughness and resistance will increase, which will lead to a significant increase in fuel consumption, thereby increasing greenhouse gas emissions [2] and indirectly increasing the rate of global warming [3]. Simultaneously, marine organisms are introduced into nonnative environments through ship transportation [4], resulting in the transfer of invasive species, which not only causes damage to the ecological environment [5] but also affects economic development and navigation safety. Therefore, marine biological pollution has always been a problem for essential issues on a global scale [6,7]. To solve this problem, at first, coatings containing biocides are applied to the surface of ships. Still, such antifouling coatings use toxic and harmful materials such as amines, amides, organotin, and cuprous oxide, which have a malignant impact on the environment and accumulate in marine organisms [8]. The high use of toxic compounds has raised concerns [9,10], and such coatings have been gradually banned worldwide [3]. Various ecologically friendly antifouling coatings have been developed, such as amphiphilic antifouling coatings, protein-resistant polymers, antifouling release coatings, conductive antifouling coatings, and biomimetic antifouling coatings [11,12,13,14,15]. Among them, fouling-release coatings with low surface energy, high elasticity, and hydrophobicity have always been the focus of researchers. Typical FR coatings consist of fluoropolymers or polydimethylsiloxane (PDMS) [16], which has a soft siloxane backbone, good water stability [17], and low modulus [18]. Still, the oil resistance is unideal, it was found by research [19] that siloxane resins are dominated by CF_2_ groups, and their stain resistance is much lower than that of CF_3_ groups. Combining the high- and low-temperature resistance of silicone and the oil resistance of organic fluorine, the fluorine group acts as a hydrophobic group [20,21]. From the perspective of antifouling, the antifouling performance of the coating can be better.

The researchers’ exploration of fluorosilicone resins has mainly focused on the introduction of fluorine groups into the polysiloxane network. The patent filed by Mera [22] produced a self-curing fluorosilicone resin crosslinked with a nonfluorinated polysiloxane resin, and the surface energy was lower than that of the pure silicone coating. Stafslien et al. [23] grafted a trifluoropropyl group (CF_3_-PDMS) and polyethylene glycol pendant groups (TMS-PEG) on the network structure of polysiloxane at the concentrations of TMS-PEG and CF_3_-PDMS as variables, and incorporating these two compounds into coatings was found to enhance the surface properties of coatings.

In this paper, hydroxyl-terminated fluoropolysiloxane was selected as the primary film-forming material to prepare the new fluorosilicone coating with three components. The main chain structure of the resin is the same as that of polydimethylsiloxane, which retains the excellent properties of the main chain of siloxane. Simultaneously, CF_3_ groups were used to enhance the antifouling performance. Nonreactive silicone oil was used to improve the fouling-release performance, and inorganic powder was used to enhance the mechanical properties of the coating.

## 2. Materials and Methods

### 2.1. Materials

Hydroxyl-terminated fluoropolysiloxane (FPS, HO[CH_3_SiCH_2_CH_2_CF_3_O]_n_OH) and methyl fluorosilicone oil (MFO, Si(CH_3_)_3_O[CH_3_SiCH_2_CH_2_CF_3_O]_n_Si(CH_3_)_3_) were obtained from Shanghai Silicon Mountain Macromolecular Materials Co., Ltd. (Shanghai, China). The Brookfield viscosity of FPS is 10,000 cp. The Brookfield viscosity of MFO is 100 cp. Hydroxy-terminated polydimethylsiloxane (PDMS, HOSi(CH_3_)_2_O[Si(CH_3_)_2_O]_n_Si(CH_3_)_2_OH) was obtained from Dayi Chemical Industry Co., Ltd. (Yantai, China). Its kinematic viscosity is 10,000 mm^2^/s. Phenyl silicone oil (PSO, Si(CH_3_)_3_O[Si(CH_3_)_2_O]_n_[Si(C_6_H_5_)_2_O]_m_Si(CH_3_)_3_) was obtained from the Damao Chemical Reagent Factory. Its kinematic viscosity is 30 mm^2^/s. Tetraethyl orthosilicate (TEOS) was obtained from Shanghai Aladdin Biochemical Technology Co., Ltd. (Shanghai, China) as an analytical grade. Dibutyltin dilaurate (DBTDL) was obtained from Tianjin Kemiou Chemical Reagent Co., Ltd. (Tianjin, China) as an analytical grade.

### 2.2. Preparation of the Coating

**Figure 1:**
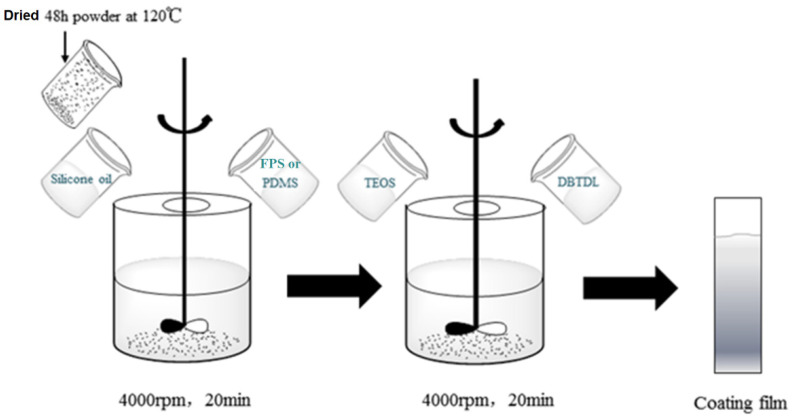
Schematic diagram of preparation process for studied coatings.

**Figure 2:**
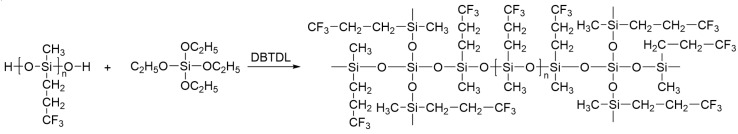
Crosslinking reaction mechanism of the fluorosilicone coatings.

The inorganic powder was dried in an oven at 120 °C for 48 h before the preparation of the paint. The coating consists of three components, namely Components A, B, and C. The formulation of Component A for prepared coatings is listed in Table 1. The preparation process of the studied coatings is shown in Figure 1. The crosslinking and curing mechanism of the fluorosilicone coatings is shown in Figure 2.

First, the primary film-forming material FPS or PDMS and xylene were placed into the BGD750 grinding, dispersing, and stirring multi-purpose machine (Guangzhou Biuged Laboratory Instruments Co., Ltd., Guangzhou, China), and nonreactive silicone oil and powder was added in sequence according to the formula proportion and was mixed at 20 min. Then, the mixture was placed into the QZM conical mill (Tianjin Jingke Material Testing Machine Factory, Tianjin, China) and ground for 30 min, obtaining Component A. Then, TEOS and Component A were mixed, stirred evenly, and matured for 30 min; DBTDL was added into the mixture and stirred for 5 min. Finally, it was brushed on glass slides with a size of 75 mm × 25 mm × 1 mm and cured for subsequent evaluations, poured into a Teflon mold of 150 mm × 150 mm × 5 mm, and cured for 7 days in ambient for tensile tests. In order to eliminate the influence of coating thickness on the results, the same weight of each paint was used to prepare these samples.

### 2.3. Methods

#### 2.3.1. Fourier Transform Infrared (FTIR) Spectroscopy

The molecular structure of the prepared coating was characterized by a Fourier Transfer Infrared Spectrometer (FTIR) (PerkinElmer Co., Ltd., Waltham, MA, USA) using the Attenuated Total Reflection (ATR) method. The scanning range was 4000–600 cm^−1^. The resolution was 2 cm^−1^. The number of scans was 32.

#### 2.3.2. Morphology Analysis 

The morphology of the coating surface was measured by an Olympus OLS4000 CLSM (OLYMPUS (China) Co., Ltd., Beijing, China), and the coating roughness (Sa) was measured by LEXT analysis software (version 2.2.4).

#### 2.3.3. Contact Angle (CA) and Surface Energy 

The water contact angle (WCA) and diiodomethane contact angle (DCA) on the studied coatings were measured using a JC2000C contact angle meter (Shanghai Zhongchen Digital Technic Apparatus Co., Ltd., Shanghai, China). The droplet size was 3 µL, and the image was taken immediately after the droplet was in contact with the coating for 3 s. The measurement calculation was performed with 6 droplets for each coating, and the average value was taken. According to the two-liquid method proposed by Owens [24], the surface free energy of each coating was calculated with Formulas (1)–(3). In the formulas, $\theta_{H_{2}O}$ is WCA; $\theta_{{\mathrm{CH}}_{2}I_{2}}$ is DCA; $\sigma_{S}^{p}$ is the polar force; $\sigma_{S}^{d}$ is the dispersion force; $\sigma_{S}$ is the surface free energy.

(1) $$\sigma_{s}^{p}=[(137.5+256.1{\;\times \cos\theta }_{H_{2}O}\;118.6{\;\times \cos\theta }_{{\mathrm{CH}}_{2}I_{2}})/44.92{]}^{2}$$

(2) $$\sigma_{s}^{d}=[(139.9+181.4{\;\times \;\cos\theta }_{{\mathrm{CH}}_{2}I_{2}}\;41.5{\;\times \cos\theta }_{H_{2}O})/44.92{]}^{2}$$

(3) $$\sigma_{s}{=\sigma }_{s}^{p}{+\sigma }_{s}^{d}$$

#### 2.3.4. Tensile Test

According to Chinese standard GB/T528-2009 (ISO37-2005) [25], the UTM5105 microcomputer-controlled electronic universal testing machine (Jinan Wance Electrical Equipment Co., Ltd., Jinan, China) was used to test the tensile properties of the samples. Three specimens were used for each coating, and the tensile speed was set to 50 mm/min. The data were analyzed to obtain the elastic modulus and stress at 100% elongation of the coating.

#### 2.3.5. Antifouling Evaluation

##### Marine Bacterial Adhesion Test

The natural seawater obtained from the Yellow Sea of Dalian in China was used for the marine bacterial adhesion test. Before the test, all utensils were sterilized in the pressure steam for 20 min, and then they were placed on the SW-CJ-1FD clean workbench for 20 min of ultraviolet disinfection.

The test process was as follows. First, 6 specimens of each coating were completely immersed in fresh seawater and incubated under 28 °C for 12 h in light and 12 h in dark. After 24 h, the samples were taken out. All 6 samples were rinsed gently in sterilized seawater to release unattached bacteria on the surface. Then, 3 samples were taken out and placed into a 50 mL centrifuge tube containing 40 mL of sterilized seawater, and the HY-4 speed-regulated multi-purpose oscillator was used to simulate the surface state of the seawater washing the coating. The oscillator parameters were as follows: vibration amplitude of 20 mm, oscillation frequency of 130 rpm, and oscillation time of 15 min. These three treated samples were named as Washed. The other three untreated ones were named as Rinsed. Then, the bacterial film was brushed on the surface of the sample into sterilized seawater and diluted 1 million times. Then, 10 µL of the diluent was taken and spread evenly on the 2216E solid medium, its formula was listed in Table 2. Next, the medium was placed into the biochemical incubator (SHP-080) and cultivated at 25 °C. It was observed once every 24 h and the number of colonies on the culture medium was recorded at 24 h and 48 h. The bacteria colony on the medium at 48 h was photographed and reported as test results. The images were quantified by Image-Pro Plus software. The average and standard deviation of the number of colonies on the three media of each group of samples were taken, and the bacterial removal rate (R) was calculated with Formula (4).

(4) $$R=\frac{{(C}_{\mathrm{Rinsed}\;}-{\;C}_{\mathrm{Washed}})}{C_{\mathrm{Rinsed}}}\;\times \;100\%$$

##### Navicula Tenera Adhesion Test

First, fresh seawater was used to prepare *Navicula Tenera* solution with an algal concentration of 10^−5^–10^−6^ cell/mL, and 6 samples were placed in the solution and cultured at 22 °C under the control of the golden ratio of 12 h:12 h. After 24 h, the samples were taken to determine the chlorophyll a value of *Navicula Tenera* attached to the coating surface. The specific methods for the culture of *Navicula Tenera* can be found in the literature [26,27]. Six samples for each coating were also treated as mentioned above and divided into rinsed and washed. To extract chlorophyll a of *Navicula Tenera* attached to the coating surface, the samples were treated as follows. Prepare 90% acetone solution, pour 45 mL into each test tube, add two drops of magnesium carbonate solution, place the rinsed and washed samples in, and extract chlorophyll for 24 h in a biochemical incubator in a dark environment at 8 °C. After completion, place 10 mL of the supernatant from each test tube into a centrifuge tube, with a centrifugation time of 15 min at a speed of 4000 rpm. Then, take 3 mL of the supernatant and place it into a cuvette, measure it using a UV-2000 UV-Vis spectrophotometer, and record the absorbance at the wavelengths of 750 nm, 663 nm, 645 nm, and 630 nm. According to Formula (5), calculate the chlorophyll a value. The diatom removal rate was similarly calculated based on the chlorophyll a concentration of the rinsed and washed samples with Formula (4).

(5) $$\rho_{a}=11.64{\;\times \;(\mathrm{OD}}_{663})\;2.16{\;\times \;(\mathrm{OD}}_{645})+0.10{\;\times \;(\mathrm{OD}}_{630})$$

## 3. Results

### 3.1. Molecular Structure

**Figure 3:**
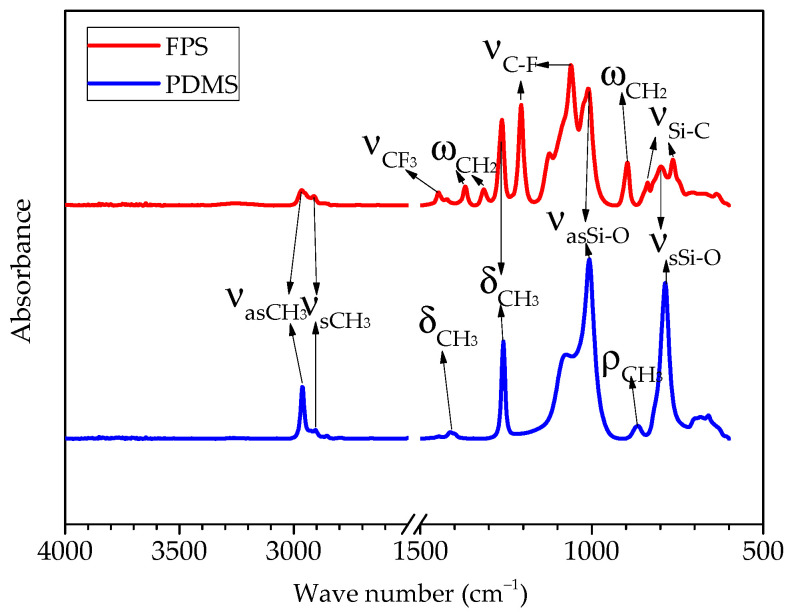
Infrared spectrum of studied coatings.

The infrared spectrum of the FPS and PDMS coating is shown in Figure 3. There was no peak during the range from 3650 to 3583 cm^−1^. This indicates that the OH group in both hydroxyl-terminated fluoropolysiloxane and polydimethylsiloxane was completely exhausted by cross-linking reaction during the curing process. For the PDMS coating, the antisymmetric stretching vibration peak was located near 2962 cm^−1^; the stretching vibration peak of the C-H bond in the CH_3_ functional group could be seen near 2905 cm^−^^1^; the symmetrical deformation vibration peak of the C-H bond appeared near 1257 cm^−1^; the antisymmetric stretching vibration peak reflected by the Si-O-Si chemical bond of the siloxane main chain appeared near 1008 cm^−1^; the characteristic peak of stretching vibration formed by the Si-O bond between the siloxane main chain and the branched-chain appeared near 785 cm^−1^. The appearance of distinct peaks indicates that the silicone coating was prepared successfully. For the FPS coating, the above-mentioned peaks were observed, and they moved to left about 3 cm^−1^. In addition, the absorption peak of CF_3_ appeared near 1446 cm^−^^1^. The characteristic peak of C-F deformation vibration appeared near 1205 cm^−1^ and 1060 cm^−^^1^, and the Si-C stretching vibration peak appeared near 837 cm^−1^ and 764 cm^−^^1^. The appearance of distinct peaks indicated that the fluorosilicone coating was successfully prepared.

### 3.2. Surface Morphology and Roughness

**Figure 4:**
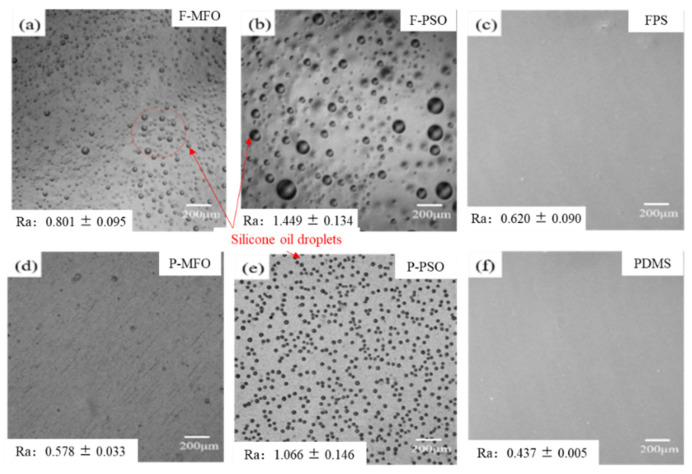
CLSM surface morphology and roughness of studied coatings: (**a**) F-MFO; (**b**) F-PSO; (**c**) FPS; (**d**) P-MFO; (**e**) P-PSO; (**f**) PDMS.

The surface morphology and roughness of the studied coatings cured for 7 days in ambient are shown in Figure 4. For the FPS and PDMS coatings without silicone oil, their surfaces looked smooth and flat. However, the surface of the coatings containing silicone oil did not look smooth, and there were many circle ridges that leached silicone oil. The roughness of the coatings with leached silicone oil was obviously higher than those of the others. This phenomenon is similar to that reported in the research [28,29,30]. They have proven that this is because of the incompatibility between nonreactive silicone oil with low molecular weight and FPS or PDMS. 

### 3.3. Interface Performance

**Figure 5:**
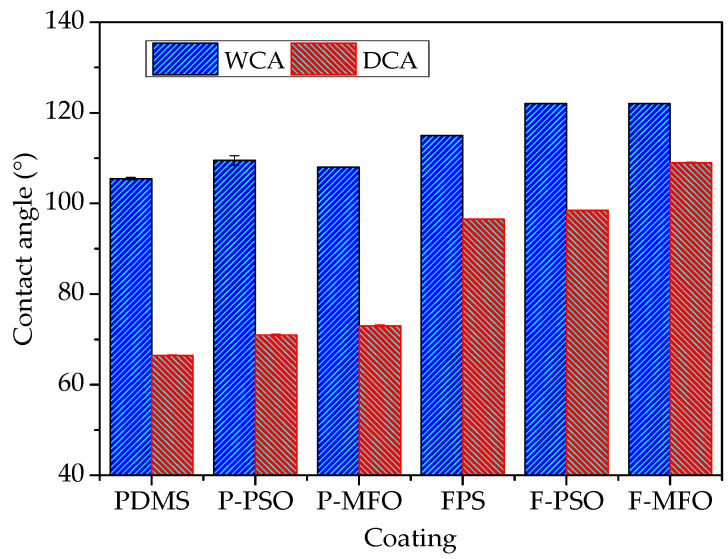
WCA and DCA of studied coatings.

**Figure 6:**
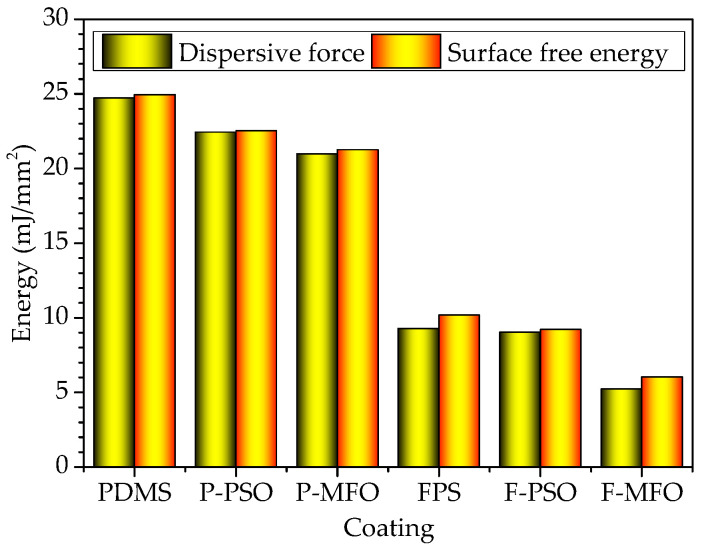
Surface energy and dispersive force of studied coatings.

The contact angle results of coatings are shown in Figure 5. The WCAs and DCAs of the FPS coatings are larger than those of the PDMS coatings. Especially, the DCAs of the FPS coatings are also larger than 90°, about 30 degrees higher than those of the PDMS coating. That indicated that the FPS coatings are not only hydrophobic but also oleophobic. The surface free energies of the FPS coatings, as shown in Figure 6, are 6.05 mJ/m^2^, 9.23 mJ/m^2^, and 10.19 mJ/m^2^, and they are much lower than the surface energy of the PDMS coating (21.26 mJ/m^2^, 22.53 mJ/m^2^, and 24.95 mJ/m^2^, respectively). This is because the outermost part of the fluorosilicone resin is densely filled with CF_3_ groups, and the polarity of the F atom is significant, which combines with the flexibility of the main chain of the Si-O bond to improve the hydrophobicity and oil repellency of the coatings, and thus reduces the surface energy of the coating. 

### 3.4. Tensile Properties 

**Figure 7:**
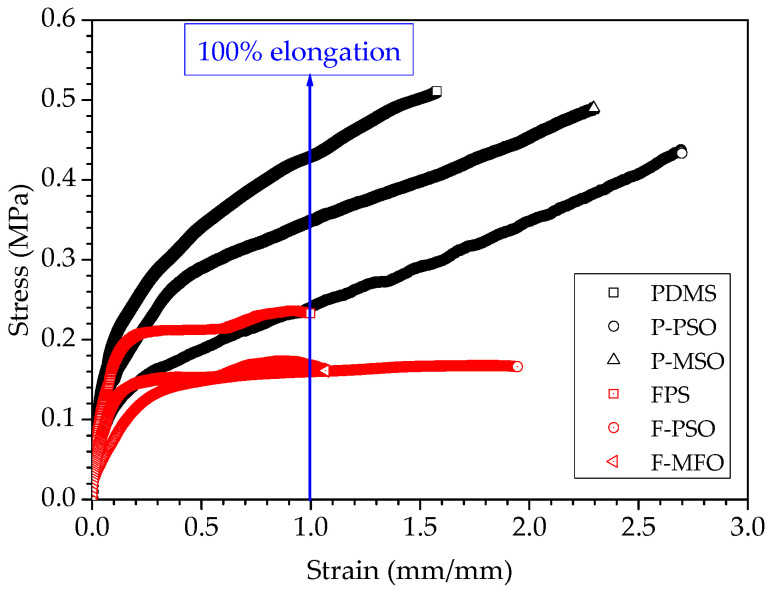
Tensile curves of studied coatings.

**Figure 8:**
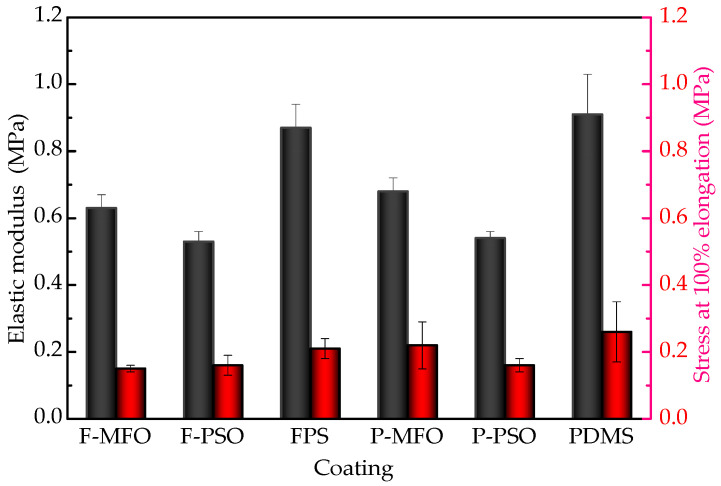
Elastic modulus and stress at 100% elongation of studied coatings.

The tensile curves and tensile properties of the studied coatings are shown in Figure 7 and Figure 8. The fracture strength and the stress at 100% elongation of the three PDMS coatings are obviously higher than those of the three FPS coatings. This can be attributed to the strengthening effect of titanium dioxide being far inferior to that of fumed silica. The elastic modulus of the coatings without silicone oil is much higher than that of the ones containing silicone oil. Introducing silicone oil can result in the reduction in the elastic modulus. Among them, the elastic modulus of the F-PSO coating is least, 0.53 MPa. This is because during the curing process of the coating, the silicone oil does not participate in the reaction and is free between the long molecular chains, decreasing the elastic modulus and stretching strength.

### 3.5. Antifouling Performance 

#### 3.5.1. Anti-Bacterial Adhesion

**Figure 9:**
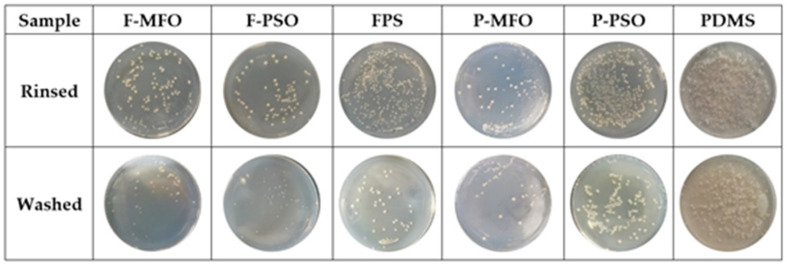
Original images of marine bacterial colonies.

**Figure 10:**
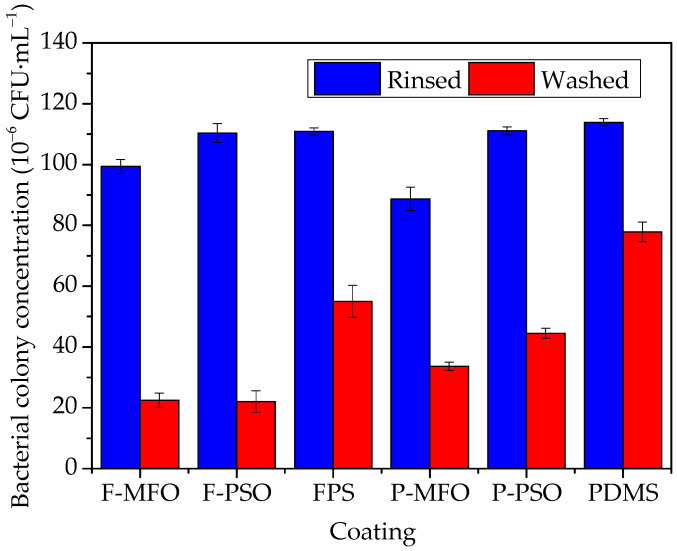
Colony concentration of bacteria attached on studied coatings.

**Figure 11:**
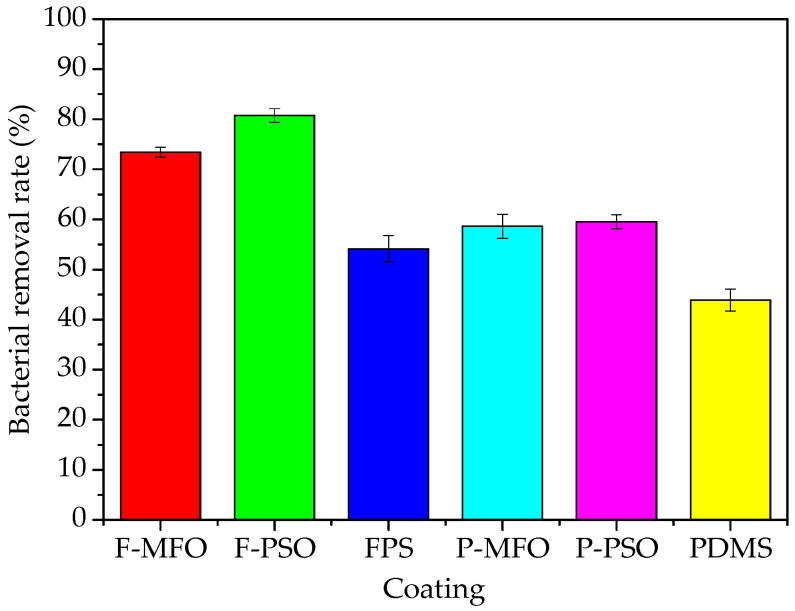
Removal rate of bacteria attached on studied coatings.

Some images of marine bacterial colonies on the solid medium are shown in Figure 9. The colony concentration and removal rates of bacteria attached on the studied coatings are shown in Figure 10 and Figure 11. Clearly, the colony concentration on washed samples is less than that on rinsed ones. In addition, that on the coatings containing silicone oil is obviously less than that on the coatings without silicone oil. The adhesive bacteria on the coatings containing silicone oil are more easily removed than that on the coatings without silicone oil. The removal rate of the coatings ranges from 50% to 80%. The removal rate of the FPS coatings is higher than that of the PDMS coatings, and that of the coatings containing silicone oil is higher than that of the ones without silicone oil. The highest is Coating F-PSO, which is 80.77%. The second is Coating F-MFO, which is 73.40%.

#### 3.5.2. Anti-Diatom Adhesion

**Figure 12:**
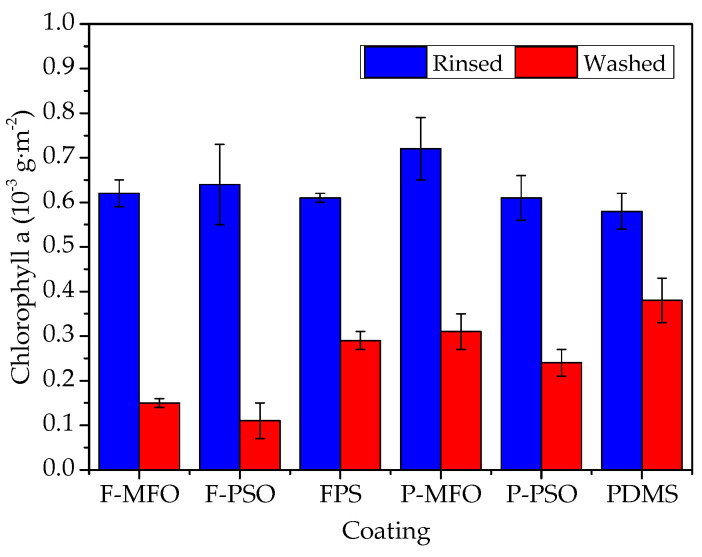
Chlorophyll a concentration of *Navicula Tenera* on studied coatings.

**Figure 13:**
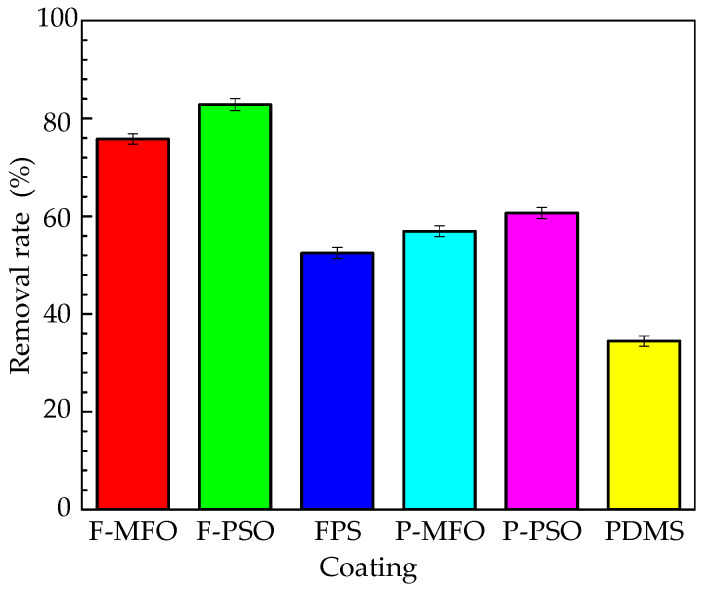
Removal rate of *Navicula Tenera* attached on studied coatings.

The Chlorophyll a concentration and removal rates of *Navicula Tenera* attached on the studied coatings are shown in Figure 12 and Figure 13. Clearly, the concentration on the washed samples is less than that on the rinsed ones. In addition, that on the coatings containing silicone oil is obviously less than that on the coatings without silicone oil. The adhesive *Navicula Tenera* on the coatings containing silicone oil is more easily removed than that on the coatings without silicone oil. The removal rate of the FPS coatings is higher than that of the PDMS coatings. Coating F-PSO has the highest diatom removal rate, which can reach 82.81%, followed by Coating F-MFO, which has a removal rate of 75.81%. Meanwhile, the FPS coating without silicone oil has a diatom removal rate of 52.46%, which is comparable to the diatom removal rate of the PDMS coating containing silicone oil. 

### 3.6. Discussion

Previous studies on fouling-release coatings have shown that the antifouling performance of coatings is related to various factors such as the surface energy, roughness, and elastic modulus of coatings [31,32,33,34]. Baier established the empirical relationship between biofouling adhesion and critical surface tension, the well-known Baier curve [3]. The surface morphology of the coating affects the attachment of marine organisms and the detachment of foulants, and the hydrophobic surface with a high roughness has a better interface performance [28]. The more the biofilms adhere to the washed samples, the worse the performance of the antifouling coating in the actual use of the marine environment [30]. Researchers have found that the wettability [31], surface chemistry [32], and surface lubricity [33] of the coating surface have a significant effect on diatom adhesion [34]. Brady [35] proposed that the relative adhesion of silicone fouling-release coatings is to the power of 1/2 of the product of elastic modulus E and surface free energy γ, i.e., Brady’s equation $Ar=\sqrt{E\gamma }$. It is considered as an important indicator for selecting and evaluating fouling-release coatings.

**Figure 14:**
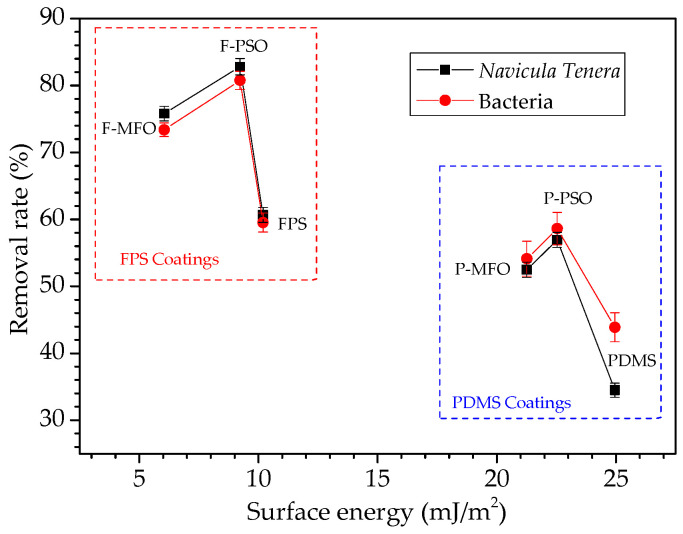
Surface energy vs. biofouling removal rate on studied coatings.

In this study, whether for bacteria or *Navicula Tenera*, its removal rate does not simply decrease with the increase in the surface energy, as shown in Figure 14. However, the removal rate of the FPS coatings is much larger than that of the PDMS coatings. Whether for FPS or PDMS coatings, the removal rate of the coatings containing silicone oil is much larger than that of the coatings without silicone oil. This is because the nonreactive low-molecular-weight silicone oil is incompatible with the matrix resin and migrates from the inside to the surface, forming a lubricating surface that increases the smoothness and hydrophobicity of the coating and decreases the surface energy [30], thereby improving the coating antifouling performance. Fluorosilicone polymer has a particular polarity and low surface energy, which can remove foulants by shearing between interfaces, and the CF_3_ group in the FPS coatings promotes the antifouling performance of the coatings. In addition, whether it is an FPS or PDMS coating, the coatings containing PSO have a higher removal rate of adhesion biofouling than that containing MFO. This is because the compatibility of PSO and the matrix resin FPS or PDMS is lower than that of MFO and the matrix resin FPS or PDMS, and phase separation is more likely to occur; PSO is also easier to migrate to the coating surface than MFO is. As shown in Figure 4, more and larger PSO droplets are observed than MFO droplets, which weakens the binding force between the attached organisms and the coating. Therefore, the attached organisms are easier to remove by the seawater washing, and the removal rate of the coating F-PSO is higher than that of the coating F-MFO.

**Figure 15:**
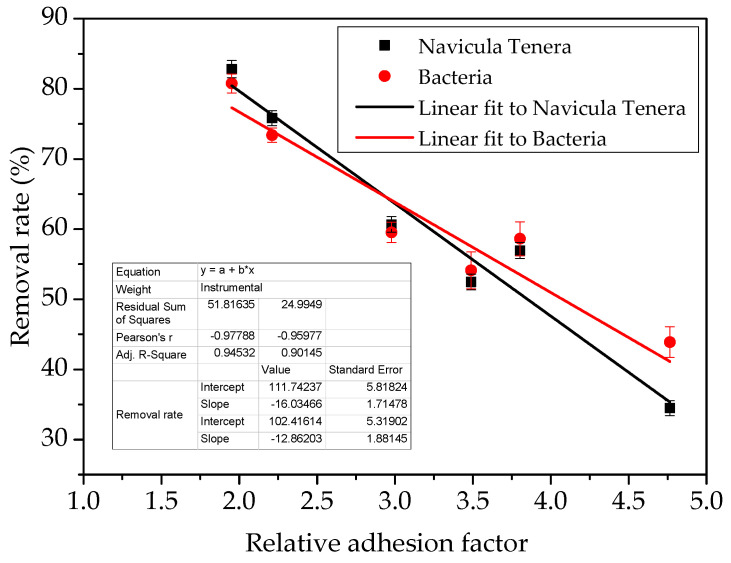
Removal rate of biofouling and relative adhesion of studied coatings.

According to Brady’s equation, the removal rates of biofouling and the relative adhesion of the studied coatings are reported in Figure 15. Obviously, whether for FPS or PDMS coatings, both the marine bacteria and *Navicula Tenera*, its removal rate linearly increases with the decrease in Brady’s relative adhesion.

**Figure 16:**
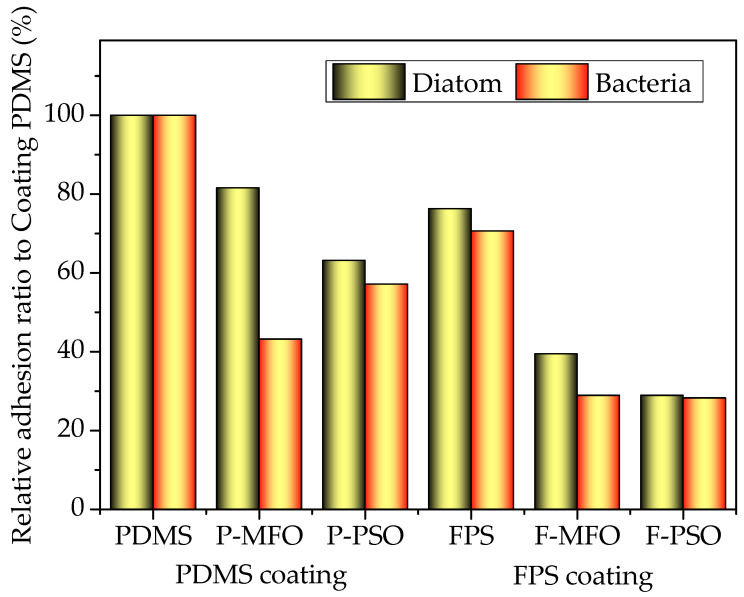
Relative ratio of attached organism on studied coating to PDMS coating.

In order to better compare the antifouling properties of coatings, the relative ratio of the colony concentration of bacteria attached and chlorophyll a concentration of *Navicula Tenera* on each washed coating to the washed PDMS coating was calculated and is reported in Figure 16. Clearly, whether for bacteria or *Navicula Tenera*, the relative ratio of the FPS coatings is much less than that of the PDMS coatings. Whether for FPS or PDMS coatings, the relative ratio of the coatings containing silicone oil is much less than that of the coatings without silicone oil. Consequently, the prepared fluorosilicone fouling-release coatings showed much better antifouling performance than PDMS coatings in this study and good application prospects. Furthermore, introducing nonreactive silicone oil into PDMS or FPS coatings can further improve the antifouling performance of the coating to a certain extent.

## 4. Conclusions

In this paper, fluorosilicone coatings (FPS) were prepared using hydroxyl-terminated fluoropolysiloxane as the primary film-forming material, TEOS as the curing agent, and DBTDL as the catalyst. The FPS coatings have a larger WCA and smaller surface free energy than that of the PDMS coatings. The FPS coatings exhibited better hydrophobic and oleophobic properties. The elastic modulus and tensile strength of the FPS coatings are lower than those of the PDMS coatings. The FPS coatings have better antifouling performance than the PDMS coatings. The prepared fluorosilicone fouling-release coatings show good application prospects. Introducing nonreactive silicone oil into PDMS or FPS coatings can improve the antifouling performance of the coating to a certain extent.

## Figures and Tables

**Table 1:** Formulation of studied coatings (g).

**Coating**	**Component**	**A**							**B**	**C**
	**Material**	**FPS**	**PDMS**	**PSO**	**MFO**	**TiO_2_**	**SiO_2_**	**Xylene**	**TEOS**	**DBTDL**
	**F-MFO**	100			5	20		25	7.5	0.4
	**F-PSO**	100		5		20		25	7.5	0.4
	**FPS**	100				20		30	7.5	0.4
	**P-MFO**		100		5	10	10	25	15	0.4
	**P-PSO**		100	5		10	10	25	15	0.4
	**PDMS**		100			10	10	30	15	0.4

**Table 2:** Formula of 2216E solid medium.

Ingredients	Peptone	Yeast Extract	FePO_4_	Agar	Sterilized Seawater
**Content**	2 g	0.4 g	0.004 g	8 g	400 mL

## Data Availability

The data in this study are contained within the article.
